# Association between paternal age and singleton birthweight in frozen embryo transfer cycles

**DOI:** 10.1186/s12978-021-01250-4

**Published:** 2021-11-03

**Authors:** Zhexin Ni, Demeng Xia, Shuai Sun, Danying Zhang, Yanping Kuang, Chaoqin Yu

**Affiliations:** 1 Department of Traditional Chinese Gynecology, Changhai Hospital Affiliated with Naval Medical University, Shanghai, China; 2 The Chinese People’s Liberation Army 91666 troops, Zhoushan, China; 3 Department of Assisted Reproduction, Shanghai Ninth People’s Hospital of JiaoTong University School of Medicine, Shanghai, China

**Keywords:** Assisted reproductive technology, Frozen embryo transfer, Vitrification, Paternal age, Birthweight

## Abstract

**Background:**

Many studies have considered maternal age as a determinant factor for success in assisted reproductive technologies (ART), but the potential role of paternal age on neonatal outcomes has been overlooked. This study aimed to explore the association between paternal age and birthweight in frozen embryo transfer (FET) cycles.

**Methods:**

This retrospective study involved singleton live births born to women undergoing frozen embryo transfer from January 2013 to December 2017 at a tertiary care center in Shanghai, China. The paternal age was classified into four categories: ≤ 30, 31–35, 36–40, and ≥ 41 years. The group consisting of respondents with paternal age of 31–35 was set as the reference group. Singleton birthweight was the primary outcome measure. Z-scores were calculated according to gestational age and newborn gender on birthweight based on the national birthweight reference. Multivariable linear regression analysis was performed to reveal the relationship between paternal age and newborns’ birthweight after considering several potential confounders.

**Results:**

Exactly 9765 women who fulfilled the inclusion criteria were enrolled. No significant difference was found on mean birthweight (*P* = 0.082) and gestation-adjusted Z-scores (*P* = 0.569) among paternal age categories. The reference group and the group with aged 36–40 years had the highest mean birthweight and Z-scores, respectively (3350.2 ± 467.8 g, 0.36 ± 1.00). A decline in mean birthweight with paternal age was observed, and the group over 40 years had the lowest value of 3309.4 ± 474.3 g, but the difference was not statistically significant. In multivariate analyses, the adjusted odds of very low birthweight (LBW), LBW, and high birthweight in the reference group did not significantly differ with the three other groups. After correcting several potential confounders, no significant correlation was observed between paternal age and neonatal birthweight (*P* = 0.289).

**Conclusion:**

Paternal age was not associated with mean birthweight and gestational age- and gender-adjusted birthweight (Z-scores) of singletons among women who became pregnant in FET cycles.

## Plain English summary

Currently, many couples delay childbearing until after the age of 40. The natural decline of fertility of elderly couples forces them to utilize assisted reproductive technology. Although males continuously produce sperm throughout their entire life, their sperm quality decreases, changes occur in reproductive hormones, the incidence of de novo mutations increases, and negative behavioral choices could cause a decline in males’ fertility potential after the age of 40. Due to their age women may also face fertility issues that have an impact on pregnancy and childbirth.

This study only included couples who had successfully given birth in frozen embryo transfer cycles. Considering that birthweight is an important indicator of neonatal health, we explored the association between paternal age and singleton birthweight. Our study found that paternal age is not associated with mean birthweight and gestational age- and gender-adjusted birthweight (Z-scores) of singletons in FET cycles, showing chances of successful pregnancy in couples over 40 years of age have improved.

## Background

Assisted reproductive technology (ART) is an effective method of ensuring person’s right to maternity or paternity. Many studies have considered maternal age as a determinant factor for success in ART [1–3], but the potential role of paternal age on neonatal outcomes has been overlooked. Older age, in women, affects neonatal outcomes, such as miscarriage, preterm birth (PTB) and birthweight [4, 5]. Although males continuously produce sperm throughout their entire life, their sperm quality decreases [6], their reproductive hormones change [7], the incidence of de novo mutations increases [8], and negative behaviors such as substance and alcohol use and smoking [9] could cause a decline in males’ fertility potential after the age of 40 [10, 11].

ART treatment aims at having a healthy baby, and a proper birthweight is an important index for the development of the fetus [12]. The birthweight of singleton newborns is closely associated with mother’s physical condition in vitrified-warmed transfer cycles, such as endometrial thickness [13], the number of oocytes retrieved [14], maternal age [15], and body mass index (BMI) [16]. However, limited information is available about the effect of paternal age on singleton newborns’ birthweight in vitrified-thawed blastocyst transfer cycles.

To our best knowledge, limited studies, have explored the effect of paternal age on ART outcomes [7, 17–20]. Only two studies have revealed the associations between paternal age and neonatal birthweight in ART, but confounders were not considered [21, 22]. However, these published papers did not rule out the possibility of adverse fetal growth resulting from a hypoestrogenic milieu because of fresh in vitro fertilization (IVF) cycles. A suboptimal peri-implantation environment for implantation and placentation can be formed by supraphysiological estrogen levels during ovarian stimulation, resulting in adverse bad effects on fetal growth [23, 24]. Unlike fresh ovarian stimulation cycles, frozen embryo transfer (FET) can provide a more physiological uterine environment for early fetal development [25]. Therefore, the current study aimed to explore the associations of paternal age with birthweight in vitrified-warmed transfer cycles in a tertiary care center from January 2013 to December 2017.

## Methods

### Study procedures

The data were obtained from the ART database with the help of the chief physician at the Department of Assisted Reproduction of the Shanghai Ninth People’s Hospital, affiliated with Jiao Tong University, School of Medicine. Details of the ART treatment and any birth resulting from ART were recorded in this database as required by the Technical Standard for Human-Assisted Reproduction issued by the Chinese Ministry of Health. This study was approved by the institutional review board of the Ninth People’s Hospital of Shanghai Jiao Tong University School of Medicine (reference number 2017-211) and was carried out in accordance with the Helsinki Declaration. Considering the retrospective nature, informed consent was not required, and patients’ data were used anonymously. To use these data reasonably, we also strictly followed the requirements of the ethics committee of Changhai Hospital of Naval Medical University (reference number CHEC 2019-100). Data stored in a computer were strictly confidential and cannot be copied away. Under the premise of not involving the privacy of patients, the data were processed and analyzed by residents with statistical training.

### Study design and patient

The retrospective study involving women who underwent FET was conducted at the Department of Assisted Reproduction of the Ninth People’s Hospital of Shanghai Jiao Tong University School of Medicine from January 2013 to December 2017. The inclusion criteria were BMI < 30 kg/m^2^, transfer of blastocyst that resulted in a live singleton birth, self-donated egg, and sperm acceptance from husband. We excluded women with vanishing twin syndrome, congenital uterine malformations as determined by ultrasound or hysteroscopy, gestational diabetes, pregnancy-induced hypertension, and preeclampsia, because these pregnancy-related factors may affect early fetal growth. If the same women had more than one delivery during the study period, only the first-born was retained.

### Laboratory protocols

The details of the procedures for ovarian stimulation, oocyte retrieval and IVF/ICSI can be found in previous studies [26, 27]. In short, IVF or ICSI was conventionally carried out based on semen parameters and previous fertilization histories. In IVF, oocytes were inseminated in human tubal fluid (HTF; Irvine Scientific) with approximately 300,000 progressively motile spermatozoa, and were supplemented with 10% (*v*/*v*) serum substitute supplement (SSS; Irvine Scientific). In ICSI, oocytes were transferred into dishes immediately after microinjection with HTF + 10% SSS. Fertilization was assessed 16–18 h post-insemination/injection, and then a dish containing preequilibrated culture medium was prepared for the transfer of zygotes. Blastocysts were cultured in a continuous single culture medium (Irvine Scientific).

All embryos were cultured under mineral oil and grew in the incubator at 37 °C with 5% O_2_ and 6% CO_2_. The embryo development was assessed on day 3, and only high-quality cleavage-stage embryos (at least six blastomeres with ≤ 20% fragmentation) were selected for cryopreservation. No change was made for the laboratory conditions and IVF protocols throughout the study period.

### Endometrial preparation and vitrification

Previous endometrial priming protocols for FET were employed [28]. By using hCG as the triggering ovulation, a natural cycle FET was selected for women with regular menstrual cycles; artificial cycles were offered for women with irregular cycles according to the discretion of attending physicians. Vitrification and thawing were conducted as previously described [27]. In short, Cryotop carrier system with dimethylsulfoxide–ethylene glycol–sucrose was employed as cryoprotectants for embryo vitrification. Dilution solution in a sequential manner (1 to 0.5 mol/L to 0 mol/L sucrose) was applied for the thawing of embryos. All embryos were thawed on the day of transfer.

### Outcome measures

Live singleton birthweight was the main neonatal outcome. The birthweight of infant was divided into the following categories: low birthweight (LBW, < 2500 g), very LBW (< 1500 g), high birthweight (HBW, > 4500 g), and normal birthweight (2500–4500 g). Live singleton birth was defined as the delivery of a singleton viable infant after the 24th gestational week.

Gestational age, Z-scores, and birthweight percentiles were the secondary outcomes. Gestational age (GA) in FET cycles was calculated from the day of embryo transfer (day 17 for cleavage stage embryo transfer) [29]. PTB and very PTB were defined as the delivery before 37 and 32 completed gestational weeks, respectively. Z-scores were calculated according to gestational age and newborn gender on birthweight based on the national birthweight reference as previously described [13]. The neonatal parameters included birthweight percentiles also based on the national birthweight reference [30]: small for GA (SGA) defined as birthweight < 10th percentile, very SGA for birthweight < 3rd percentile, large for GA (LGA) defined as birthweight > 90th percentile, and very LGA for birthweight > 97th percentile. Data regarding pregnancy and neonatal outcomes were obtained from electronic medical records. Missing data were checked by contact with midwives or treating obstetricians.

### Exposure variable and adjustment variables

Paternal age at the birth of the child was the key exposure variable in the present study, and was categorized as follows: aged ≤ 30, 31–35, 36–40, and ≥ 41 years. Paternal age of 31–35 was set as the reference category in our analyses. The adjustment variables included maternal age, paternal education, maternal education, maternal BMI, infertility cause, infertility duration, parity, FET cycle rank, fertilization method, the type of endometrial preparation, endometrial thickness, number of embryos transferred, embryo developmental stage at transfer, delivery mode, and newborn gender.

### Statistical analysis

One-way analysis of variance was performed for continuous data, while Pearson’s chi-squared test was applied for categorical data. The association between paternal age and neonatal outcomes was determined using multivariable logistic regression analysis, while the independent effect of paternal age on neonatal outcomes was analyzed using multiple linear regression. Univariate and multivariate logistic regression were performed for the outcomes of PTB, early PTB, LBW, very LBW, HBW, SGA, very SGA, LGA, and very SGA. The crude odds ratios (OR) and adjusted OR (aOR) with 95% confidence interval (CI) for the respective outcomes were calculated. The paternal age was categorized into ≤ 30, 31–35, 36–40, and ≥ 41. The interval of 31–35 years was used as a reference, while the three other groups were included in the logistic regression analysis as dummy variables. The factors above, as adjustment variables, were initially included as potential confounders. According to the results of univariate and stepwise regression combined with factors that were known or suspected to be related to clinical outcome, we finally performed multivariable regression. In multivariable models, continuous covariates (paternal age, maternal age, maternal BMI, infertility duration, endometrial thickness and gestational age) and categorical covariates are summarized in Table 1. All analyses were conducted in SPSS Statistics (version 21.0), and P < 0.05 was considered to be statistically significant.

**Table 1:** Patient treatment and demographic characteristics according to paternal age

	≤ 30 years n = 2634	31–35 years n = 3886	36–40 years n = 2129	≥ 41 years n = 1116	*P* value
Maternal age (year)	28.07 ± 2.58	31.66 ± 2.62	34.91 ± 2.93	37.77 ± 3.67	< 0.001^a^
Maternal BMI (kg/m^2^)	21.22 ± 2.60	21.35 ± 2.56	21.66 ± 2.58	21.79 ± 2.48	< 0.001^a^
Paternal education					< 0.001^b^
Below high school	565 (21.5)	507 (13.0)	345 (16.2)	170 (15.2)	
High school	459 (17.4)	440 (11.3)	276 (13.0)	173 (15.5)	
College	1553 (59.0)	2570 (66.1)	1281 (60.2)	660 (59.1)	
Above college	57 (2.2)	369 (9.5)	227 (10.7)	113 (10.1)	
Maternal education					< 0.001^b^
Below high school	642 (24.4)	628 (16.2)	371 (17.4)	209 (18.7)	
High school	427 (16.2)	456 (11.7)	281 (13.2)	160 (14.3)	
College	1525 (57.9)	2522 (64.9)	1303 (61.2)	655 (58.7)	
Above college	40 (1.5)	280 (7.2)	174 (8.2)	92 (8.2)	
Infertility cause					< 0.001^b^
Female	1936 (73.5)	2857 (73.5)	1531 (71.9)	771 (69.1)	
Male	342 (13.0)	408 (10.5)	208 (9.8)	133 (11.9)	
Mixed	319 (12.1)	520 (13.4)	325 (15.3)	188 (16.8)	
Unexplained	37 (1.4)	101 (2.6)	65 (3.1)	24 (2.2)	
Infertility duration (years)	2.44 ± 1.83	3.16 ± 2.35	4.00 ± 3.28	4.18 ± 4.01	< 0.001^a^
Parity					< 0.001^b^
0	2556 (97.0)	3681 (94.7)	1885 (88.5)	906 (81.2)	
> 0	78 (3.0)	205 (5.3)	244 (11.5)	210 (18.8)	
FET cycle rank					< 0.001^b^
First	1703 (64.7)	2240 (57.6)	1179 (55.4)	589 (52.8)	
High order	931 (35.3)	1646 (42.4)	950 (44.6)	527 (47.2)	
FET endometrial preparation					0.001^b^
Natural cycle	539 (20.5)	898 (23.1)	529 (24.8)	282 (25.3)	
Artificial cycle	2095 (79.5)	2988 (76.9)	1600 (75.2)	834 (74.7)	
Endometrial thickness (mm)					< 0.001^b^
< 8	178 (6.8)	277 (7.1)	190 (8.9)	132 (11.8)	
8–11	1479 (56.2)	2199 (56.6)	1222 (57.4)	627 (56.2)	
> 11	977 (37.1)	1410 (36.3)	717 (33.7)	357 (32.0)	
Fertilization method					< 0.001^b^
IVF	1614 (61.3)	2463 (63.4)	1309 (61.5)	670 (60.0)	
ICSI	729 (27.7)	1002 (25.8)	613 (28.8)	386 (34.6)	
IVF + ICSI	291 (11.0)	421 (10.8)	207 (9.7)	60 (5.4)	
Number of embryos transferred					0.583^b^
1	2232 (84.7)	3272 (84.2)	1821 (85.5)	942 (84.4)	
≥ 2	402 (15.3)	614 (15.8)	308 (14.5)	174 (15.6)	
Embryo developmental stage at transfer					0.003^b^
Day 3	427 (16.2)	694 (17.9)	407 (19.1)	234 (21.0)	
Day 5/6	2207 (83.8)	3192 (82.1)	1722 (80.9)	882 (79.0)	
Delivery mode					< 0.001^b^
Natural birth	845 (32.1)	1139 (29.3)	504 (23.7)	219 (19.6)	
Cesarean section	1789 (67.9)	2747 (70.7)	1625 (76.3)	897 (80.4)	

Data are mean ± S.D. or n (%)

^a^One-way ANOVA

^b^Pearson chi-square test

## Results

### Participants sociodemographic characteristics

A total of 9765 women who met the inclusion criteria were included in the analysis, and their baseline demographic and cycle characteristics are presented in Table 1. Significant differences were demonstrated based on the comparison among paternal age categories on maternal age (*P* < 0.001), maternal BMI (*P* < 0.001), paternal education (*P* < 0.001), maternal education (*P* < 0.001), infertility cause (*P* < 0.001), infertility duration (*P* < 0.001), parity (*P* < 0.001), FET cycle rank (*P* < 0.001), FET endometrial preparation (*P* < 0.001), endometrial thickness (*P* < 0.001), fertilization method (*P* < 0.001), embryo developmental stage at transfer (*P* = 0.003) and delivery mode (*P* < 0.001). The number of embryos transferred did not differ significantly among the four groups.

### Perinatal outcomes by paternal age

Table 2 shows the neonatal outcomes stratified by paternal age. The mean gestational age significantly declined (*P* < 0.001), whereas no significant difference was found on mean birthweight (*P* = 0.082) and gestation-adjusted Z-scores (*P* = 0.569) among paternal age categories. The reference group and the group with aged 36–40 years had the highest mean birthweight and Z-scores, respectively (3350.2 ± 467.8 g, 0.36 ± 1.00). A decreased decline of mean birthweight with paternal age was observed, and the group over 40 years had the lowest value of 3309.4 ± 474.3 g, but the difference was not statistically significant. The highest proportion of LBW (4.2%) was found in the group aged over 40 years, while the highest proportion of HBW (6.8%) was observed in the group under aged 30 years, and no significant difference was observed. Furthermore, no significant difference was observed in gestational age grouping, birthweight for gestational age and newborn gender among four groups.

**Table 2:** Neonatal outcomes of live born singletons sorted by paternal age

	≤ 30 years n = 2634	31–35 years n = 3886	36–40 years n = 2129	≥ 41years n = 1116	*P* value
Gestational age (week)	38.62 ± 1.51	38.64 ± 1.41	38.50 ± 1.52	38.36 ± 1.41	< 0.001^a^
Gestational age grouping					0.461^b^
Very preterm birth (< 32 weeks)	18 (0.7)	15 (0.4)	14 (0.7)	7 (0.6)	
Preterm birth (< 37 weeks)	132 (5.0)	218 (5.6)	128 (6.0)	63 (5.6)	
≥ 37 weeks	2484 (94.3)	3653 (94.0)	1987 (93.3)	1046 (93.7)	
Birthweight (g)	3345.0 ± 480.7	3350.2 ± 467.8	3337.4 ± 481.1	3309.4 ± 474.3	0.082^a^
Z-scores	0.34 ± 1.01	0.34 ± 1.02	0.36 ± 1.00	0.30 ± 1.01	0.569^a^
Birthweight grouping					0.176^b^
Very low birthweight (< 1500 g)	10 (0.4)	7 (0.2)	9 (0.4)	5 (0.4)	
Low birthweight (< 2500 g)	77 (2.9)	119 (3.1)	65 (3.1)	47 (4.2)	
Birthweight (2500–4000 g)	2367 (89.9)	3524 (90.7)	1933 (90.8)	1005 (90.1)	
High birthweight (> 4000 g)	180 (6.8)	236 (6.1)	122 (5.7)	59 (5.3)	
Birthweight for gestational age					0.282^b^
Very small for gestational age (< 3rd percentile)	35 (1.3)	55 (1.4)	22 (1.0)	16 (1.4)	
Small for gestational age (< 10th percentile)	88 (3.3)	129 (3.3)	70 (3.3)	32 (2.9)	
Birthweight for gestational age (10th–90th)	2087 (79.2)	3052 (78.5)	1678 (78.8)	893 (80.0)	
Large for gestational age (> 90th percentile)	242 (9.2)	419 (10.8)	236 (11.1)	121 (10.8)	
Very large of gestational age (> 97th percentile)	182 (6.9)	231 (5.9)	123 (5.8)	54 (4.8)	
Newborn gender					0.777^b^
Female	1373 (52.1)	2006 (51.6)	1100 (51.7)	595 (53.3)	
Male	1261 (47.9)	1880 (48.4)	1029 (48.3)	521 (46.7)	

Data are mean ± S.D. or n (%)

^a^One-way ANOVA

^b^Pearson chi-square test

Although no significant difference was found on birthweight among four paternal age groups, several adjustment variables have reached significant difference. To eliminate the influence by the confounders and determine the real risks of abnormal birthweight by paternal age, we conducted multivariate analyses (Table 3). Results showed that the odds of LBW, very LBW, and HBW in the reference group did not have significant difference compared with those of the three other groups. The risk of preterm birth (< 37 weeks) was slightly increased in the group under the age of 30 compared with those in the reference group (aOR 1.157, 95% CI 0.887–1.510). The odds of very SGA (< 3rd percentile), SGA (< 10th percentile), LGA (> 90th percentile), and very LGA (> 97th percentile) was higher in group aged over 40 years compared with the reference group, but significant difference was not observed.

**Table 3:** Odds ratios for gestational age and birth weights by paternal age

	≤ 30 years n = 2634	31–35 years n = 3896	36–40 years n = 2129	≥ 41 years n = 1116
Gestational age < 32 weeks				
Crude OR	0.567 (0.285, 1.126)	Reference	0.583 (0.281, 1.210)	0.614 (0.250, 1.509)
Adjusted OR	0.602 (0.263, 1.377)	Reference	0.676 (0.285, 1.603)	0.971 (0.298, 3.160)
Gestational age < 37 weeks				
Crude OR	1.123 (0.899, 1.402)	Reference	0.926 (0.740, 1.160)	0.991 (0.742, 1.322)
Adjusted OR	1.157 (0.887, 1.510)	Reference	0.905 (0.700, 1.171)	0.980 (0.676, 1.419)
Very low birthweight (< 1500 g)				
Crude OR	0.470 (0.179, 1.237)	Reference	0.427 (0.159, 1.147)	0.399 (0.126, 1.261)
Adjusted OR	0.373 (0.117, 1.191)	Reference	0.503 (0.164, 1.544)	0.459 (0.114, 1.848)
Low birthweight (< 2500 g)				
Crude OR	1.038 (0.776, 1.389)	Reference	1.004 (0.739, 1.366)	0.722 (0.511, 1.019)
Adjusted OR	0.974 (0.690, 1.375)	Reference	1.076 (0.755, 1.533)	0.952 (0.596, 1.521)
High birthweight (> 4000 g)				
Crude OR	0.881 (0.720, 1.077)	Reference	1.061 (0.847, 1.329)	1.141 (0.850, 1.530)
Adjusted OR	1.013 (0.792, 1.294)	Reference	1.040 (0.803, 1.348)	1.141 (0.784, 1.660)
Very small for gestational age (< 3rd percentile)				
Crude OR	1.075 (0.701, 1.648)	Reference	1.375 (0.835, 2.262)	1.006 (0.574, 1.764)
Adjusted OR	1.013 (0.858, 1.165)	Reference	1.304 (0.739, 2.300)	1.101 (0.513, 2.362)
Small for gestational age (< 10th percentile)				
Crude OR	1.002 (0.760, 1.322)	Reference	1.013 (0.753, 1.364)	1.180 (0.795, 1.749)
Adjusted OR	1.069 (0.760, 1.505)	Reference	1.107 (0.791, 1.550)	1.409 (0.859, 2.312)
Large for gestational age (> 90th percentile)				
Crude OR	1.184 (1.001, 1.400)	Reference	0.976 (0.823, 1.157)	1.013 (0.817, 1.257)
Adjusted OR	1.220 (0.995, 1.495)	Reference	1.003 (0.826, 1.218)	1.068 (0.811, 1.407)
Very large of gestational age (> 97th percentile)				
Crude OR	0.868 (0.709, 1.062)	Reference	1.033 (0.823, 1.295)	1.252 (0.922, 1.699)
Adjusted OR	0.928 (0.724, 1.188)	Reference	1.054 (0.812, 1.368)	1.379 (0.934, 2.036)

Data are odds ratios (OR) with 95% confidence interval (CI). Analyses were adjusted for maternal age, paternal education, maternal education, maternal BMI, infertility cause, infertility duration, parity, FET cycle rank, fertilization method, the type of endometrial preparation, endometrial thickness, number of embryos transferred, embryo developmental stage at transfer, delivery mode and newborn gender

The adjustment variables such as parents’ education, infertility cause and FET endometrial preparation may affect the association between paternal age and birthweight. We performed multiple linear regression analyses to assess the associations between paternal age and birthweight (Table 4), and it was corrected for several potential confounders. No significant correlation was observed between paternal age and neonatal birthweight. However, maternal BMI (*P* < 0.001), parity (*P* = 0.005), FET cycle rank (*P* = 0.018), endometrial thickness (8–11 mm, *P* = 0.029; > 11 mm, *P* = 0.003), embryo developmental stage at transfer (*P* < 0.001), and gestational age (*P* < 0.001) were all independent predictors for birthweight.

**Table 4:** Results of multiple regression analysis of singleton birthweight

	Unstandardised coefficients	Std. error	Standardised coefficients	t	*P* value
	B		Beta		
(Constant)	− 3953.883	118.402		− 33.394	< 0.001
Paternal age (year)	1.224	1.154	0.014	1.060	0.289
Paternal education					
Below high school	− 23.543	16.245	− 0.018	− 1.449	0.147
High school (reference)					
College	− 16.949	13.792	− 0.017	− 1.229	0.219
Above college	− 40.042	21.485	− 0.023	− 1.864	0.062
Maternal age (year)	− 1.714	1.524	− 0.015	− 1.125	0.261
Maternal BMI (kg/m^2^)	24.929	1.562	0.135	15.959	< 0.001
Maternal education					
Below high school	− 13.133	15.866	− 0.011	− 0.828	0.408
High school (reference)					
College	− 9.940	13.791	− 0.010	− 0.721	0.471
Above college	− 6.272	23.371	− 0.003	− 0.268	0.788
Infertility cause					
Female (reference)					
Male	− 16.308	14.775	− 0.011	− 1.104	0.270
Mixed	− 11.251	12.576	− 0.008	− 0.895	0.371
Unexplained	33.220	26.724	0.011	1.243	0.214
Infertility duration (years)	0.149	1.528	0.001	0.098	0.922
Parity, high order (versus 0)	44.461	15.988	0.025	2.781	0.005
FET cycle rank, high order (versus first)	19.885	8.380	0.021	2.373	0.018
FET endometrial preparation, artificial cycle (versus natural cycle)	− 15.151	9.444	− 0.013	− 1.604	0.109
Endometrial thickness (mm)					
< 8 (reference)					
8–11	32.929	15.073	0.034	2.185	0.029
> 11	46.007	15.670	0.046	2.936	0.003
Fertilization method					
IVF (reference)					
ICSI	− 17.118	10.853	− 0.016	− 1.577	0.115
IVF + ICSI	0.574	13.917	< 0.001	0.041	0.967
Number of embryos transferred, ≥ 2 (versus 1)	14.579	11.651	0.012	1.251	0.211
Embryo developmental stage at transfer, day 5/6 (versus day 3)	70.051	12.674	0.053	5.527	< 0.001
Gestational age (weeks)	176.728	2.722	0.546	64.915	< 0.001
Newborn gender, female (versus male)	− 1.714	1.524	− 0.015	− 1.125	0.261

## Discussion

Recently, studies have revealed that advanced paternal age (over 40 years old) has an adverse effect on ART outcomes, such as sperm quality, pregnancy rate, and miscarriage rate [7, 17–20]. However, the association between paternal age and singleton birthweight in FET cycles has not been revealed. The present study has demonstrated that paternal age does not affect singleton newborns’ birthweight in FET cycles, and paternal age is not an independent predictor of neonatal birthweight as indicated by linear regression analysis.

In response to the aging population, China has implemented the two-child policy. On the one hand, the rapid economic development has enabled ordinary families to raise two children, prompting many couples over the age of 40 years to have a second child. On the other hand, the rapid pace of life brought by economic rise makes many young people feel more pressure and choose to postpone childbearing. Influenced by Chinese Confucian culture, men are dominant in the family, and Chinese people should have children and pass down their surnames, which will be blessed by their ancestors. In such a social environment, people may choose to consider childbearing after the family economic conditions reach a certain reliable level, and they may become more dependent on ART.

Two studies have explored the potential relationship between paternal age and neonatal birthweight. Stern et al. [21] investigated the effect of paternal age in fertile, subfertile, and ART pregnancies that involved 9092 ART cycles, suggesting that older father’s age was not associated with increased risk for prematurity, low birthweight, or SGA. However, the study did not consider known confounders and excluded the effect of a hypoestrogenic milieu because of fresh IVF cycles. Additionally, they may have missed some deliveries to couples with infertility issues caused by the conservative definition of the subfertile group. Another study [22] reported that for the singletons, none of the variables, including low birthweight, clinical pregnancy rate and abortion risk, differed among the five groups categorized by paternal age. Among the twins, however, the infants in the group with aged 36–40 year had a lower neonatal birthweight and a higher low-birthweight rate than those of the other groups. The number of involved neonates is small (n = 1310) and similar deficiencies as mentioned in the study by Stern et al. [21] were observed.

The present study mainly aimed to improve the gaps in previous studies and reveal the exact association of paternal age with the neonatal birthweight in FET cycles. The current study involved 9765 singleton newborns, indicating that paternal age itself did not affect singleton birthweight. Our study found no significant correlation between paternal age and birthweight. Notably, we only included couples who had successfully given birth, and naturally excluded many cases of failure. However, paternal age influences sperm quality with advanced paternal age, thus decreasing the volume, total sperm count, sperm motility, sperm morphology, and sperm DNA integrity [31–33]. This phenomenon may be caused by sperm DNA damage because of methylation and exposure to reactive oxygen species [34, 35]. The age of the spouse also increases with paternal age, leading to reduced in ovarian function, decreased ovarian response to ovulation promoting drugs [14], decline in the quality of oocytes [36, 37], abnormal endometrial function and degeneration of multiple organs function [13, 38]. Neonatal outcomes in ART would be affected by the abovementioned factors.

In addition, Li et al.’s study [39] shows that low paternal education and weight are associated with increased incidence of PTB and SGA. Paternal overweight is associated with decreased LBW, PTB and SGA, and paternal smoking is associated with high risk of PTB. Although these results were based on natural pregnancy, these associations could provide clues about the etiology of these conditions in FET cycles. Further scientific and epidemiologic studies are needed to elucidate the mechanism of how paternal factors influence fetal developmental processes in FET cycles. Habib et al.’s study [40] indicated that paternal social characteristics have a stronger influence on perinatal mortality than maternal characteristics in North East Tanzania, which may reflect social and cultural conditions that need to be considered by policymakers in developing countries. However, considering that educational, social, economic, and cultural resources for the public are improved in China, more people choose to have a late marriage, and therefore late childbearing. In comparison with families having young children, single men and women who prefer late marriage have more leisure to enjoy themselves and bear fewer financial burdens. Meanwhile, they can obtain more educational time and opportunities. These knowledgeable individuals have good living habits, health conditions, and economic strengths, thus having more opportunities to access spouse of corresponding level and better medical resources in ART treatment. Although aging leads to an irreversible decline in fertility, it may enhance life experience and result in wealth accumulation and a more cautious life attitude. Older couples with access to and available health services, financial independence, and good social support are likely to focus on pregnancy and seek medical help more actively than the young [1]. Moreover, we cannot ignore the development of ART, which has well fulfilled the reproductive needs of couples of different ages to improve newborns outcomes. Hence, paternal age is not associated with singleton newborns’ birthweight after FET cycles in this study.

Some literature suggests that a father’s involvement in prenatal care engenders health benefits for both mothers and children [41–43]. While this information can help practitioners improve family health, low paternal involvement in prenatal care remains a challenge. Father involvement during pregnancy is associated with increased receipt of prenatal care, reduced maternal alcohol and tobacco use, and a lower likelihood of LBW infants. Moreover, involvement during the prenatal period is a strong predictor of involvement later in the child’s life, with continued positive outcomes. Although our research lacks this part of data, we actively advocate father’s involvement in prenatal care. Considering that the elderly population is increasing and couples of childbearing ages are busy working in China, grandparents of newborns should participate in the perinatal period, thus possibly improving health benefits for both mothers and children.

In the present study, data from multiple linear regression analysis showed that maternal BMI, parity, FET cycle rank, endometrial thickness, embryo developmental stage at transfer and gestational age were all independent predictors for birthweight, as previously described [13, 16, 44]. A one-unit increase (1 kg/m^2^) in maternal BMI is associated with 24.9 g increase in newborn birthweight. The predicted weight of high order parity is 44 g higher than zero parity, while high order FET cycle rank 19 g higher than the first FET cycle rank. One millimeter increase in endometrial thickness can cause 32–46 g increase in newborn birthweight. Day 5/6 embryo transfer has been expected to weigh 70 g higher than day 3 embryo transfer. Each weekly increase in gestational age is related to a 176 g increase in birthweight.

Our study has limitations. The most obvious limitation is its retrospective design, specifically the lack of detailed perinatal data, such as semen analysis. We only included women who successfully given birth and naturally excluded many cases of failure. Considering the lack of data, we did not control paternal life-style factors, such as BMI, adiposity and cigarette smoking, and substance use. This study aimed to determine the impact of paternal age on birthweight and studies that investigated the relationship between paternal factors and adverse birth outcomes have reached inconsistent results [18, 20, 45]. Hence, further studies will be necessary to explore more detailed associations between paternal factors and neonatal outcomes. Notably, the large number of singleton live births from a single center is the main strength of the current study. All laboratory conditions and protocols remained invariant throughout the study period, and several potential confounders were included in our study to increase the credibility of the results.

## Conclusions

In summary, this study expands the current knowledge about the association between paternal age and IVF/ICSI outcomes and indicated that paternal age is not associated with mean birthweight and Z-scores. This important finding shows chances of successful pregnancy in couples aged over 40 years have improved. However, specific analysis is needed for individuals. Aside from age, social, economic, psychological, and educational problems, which may affect the ART outcomes, should be considered. Under China’s two child policy, the abovementioned factors should be balanced to achieve the best neonatal outcome. This study may provide decision-making reference for the government and thus provide social welfare preference to the people of specific age groups in FET cycles and help them improve the birth outcomes and achieve social equity in China.

## Data Availability

The data that support the findings of this study are available from the Ninth People’s Hospital of Shanghai Jiao Tong University School of Medicine, but restrictions apply to the availability of these data, which were used under guidelines for the current study, and so are not publicly available.

## Abbreviations

FET: Frozen embryo transfer
ART: Assisted reproductive technology
PTB: Preterm birth
BMI: Body mass index
IVF: In vitro fertilization
ICSI: Intracytoplasmic sperm injection
GA: Gestational age
LBW: Low birthweight
HBW: High birthweight
SGA: Small for gestational age
LGA: Large for gestational age
OR: Odds ratios
aOR: Adjusted odds ratios
CI: Confidence interval
